# Perioperative outcomes and prevalence of urethral strictures during surgery for benign prostatic hyperplasia: results from the GRAND study

**DOI:** 10.1007/s00345-026-06776-5

**Published:** 2026-09-17

**Authors:** Nikolaos Pyrgidis, Gerald Bastian Schulz, Philipp Weinhold, Yannic Volz, Michael Atzler, Leo Federico Stadelmeier, Iason Papadopoulos, Christian Stief, Julian Marcon, Patrick Keller

**Affiliations:** Department of Urology, University Hospital, LMU Munich, Marchioninistraße 15, 81377 Munich, Germany

**Keywords:** Benign prostatic hyperplasia, Laser enucleation, Transurethral resection of the prostate, Urethral stricture, Perioperative outcomes

## Abstract

**Background:**

Limited data exist on the prevalence of urethral strictures during surgery for benign prostatic hyperplasia (BPH). We aimed to analyze their perioperative outcomes and trends.

**Materials and methods:**

The German Nationwide Inpatient Data (GRAND) registry was used to identify male patients diagnosed with urethral stricture who underwent surgery for BPH between 2005 and 2022.

**Results:**

A total of 1,367,065 BPH surgeries were performed in Germany. Of them, 179,272 had a relevant concomitant urethral stricture (13%). Their median age was 72 years (interquartile range: 66–78). Patients with urethral stricture during BPH surgery underwent an internal urethrotomy in 155,482 (86%) cases, a dilatation of the urethral stricture in 14,162 (9%) cases, or a meatoplasty in 9,628 (5%) cases. The annual prevalence of relevant urethral strictures during BPH surgery has decreased from 14,872 cases in 2005 to 7,184 in 2022. Compared to patients with no urethral stricture, those with urethral strictures during BPH surgery displayed higher rates of postoperative incontinence (3.8% versus 3.7%, p<0.001) and reoperations (1.0% versus 0.8%, p<0.001). On the contrary, they were associated with lower rates of perioperative acute urinary retention (7.8% versus 10%, *p*<0.001).

**Conclusion:**

Urethral strictures during surgery for BPH display a high prevalence and may slightly affect perioperative outcomes.

## Introduction

Lower urinary tract symptoms (LUTS) secondary to benign prostatic hyperplasia (BPH) represent a highly prevalent condition among aging men and constitute a major burden on healthcare systems worldwide [1]. Surgical management remains the cornerstone of treatment for patients with moderate-to-severe LUTS refractory to medical therapy [2]. Among available options, transurethral resection of the prostate (TURP) has historically been considered the gold-standard procedure [3]. At the same time, laser-based approaches such as holmium laser enucleation of the prostate (HoLEP) or thulium laser enucleation of the prostate (ThuLEP) are increasingly used due to their favorable perioperative outcomes and efficacy in larger prostates [4]. Additionally, minimally invasive treatments have recently made their way into the clinical pipeline [5].

Despite continuous advancements in surgical techniques and instrumentation, perioperative challenges remain [6]. One clinically relevant, but often underreported, condition is the presence of concomitant urethral stricture in patients undergoing surgery for BPH [7, 8]. Urethral strictures may arise from prior instrumentation, inflammation, trauma, or other causes and can complicate transurethral access, prolong operative time, necessitate additional interventions, and, in turn, negatively affect perioperative outcomes [9]. Importantly, urethral strictures lead to LUTS, which may be falsely attributed to BPH [10]. Consequently, their presence and preoperative diagnosis are of utmost importance and may influence surgical strategies [11].

Furthermore, the development of urethral strictures after transurethral surgery has frequently been attributed to the use of standard 26-Fr resectoscopes [12]. However, it remains unclear whether these strictures truly represent a procedure-related complication or rather reflect pre-existing urethral pathology that was already present before surgery [13]. Therefore, a better understanding of the prevalence of preoperative urethral strictures and their relationship to postoperative stricture rates is warranted. Current evidence regarding urethral strictures in the context of BPH surgery is limited and predominantly derives from small, single-center studies [14]. While urethral stricture disease itself has been extensively studied, its prevalence during BPH surgery, associated management strategies, and impact on perioperative outcomes at a population level remain undercaptured [15]. In particular, there is a lack of large-scale data evaluating temporal trends in the management of urethral strictures when encountered intraoperatively, as well as their potential implications for complications such as urinary retention, incontinence, and reintervention [16]. In this context, we aimed to analyze the prevalence, temporal trends, and perioperative outcomes of urethral strictures in patients undergoing surgical treatment for BPH using the German Nationwide Inpatient Data (GRAND) registry over 17 years.

## Methods

### German nationwide inpatient data (GRAND)

This study was based on data derived from the GRAND registry, administered by the Research Data Center of the Federal Statistical Office in Wiesbaden, Germany. The registry contains anonymized information on all inpatient hospitalizations in Germany from 2005 to 2022. Admissions related to military, psychiatric, or forensic care are not included in the database. Our analyses were conducted using aggregated summary tables provided by the Research Data Center. Therefore, individual-level patient records were not directly accessible to the research team. Access to the anonymized dataset was granted following formal approval (agreement number: LMU-4710-2022). According to German regulations governing the use of fully anonymized administrative data, neither ethical committee approval nor informed patient consent was required.

Since the implementation of the Diagnosis Related Groups (DRG) reimbursement system in 2005, hospitals in Germany have to report detailed information on diagnoses, perioperative complications, and performed procedures to the Institute for the Hospital Remuneration System. Diagnostic information and complications are coded using the International Classification of Diseases, 10th Revision, German Modification (ICD-10-GM), whereas surgical procedures are documented according to the German Procedure Classification (OPS). The German Institute for Medical Documentation and Information is responsible for maintaining standardized coding practices nationwide. The German Institute for Medical Documentation and Information then transfers this information to the Research Data Center of the Federal Statistical Office in Wiesbaden, Germany for research purposes.

### Data source and study population

Male patients with concomitant BPH (ICD-10-GM code: N40) and urethral stricture (ICD-10-GM code: N35) were identified if they underwent surgical treatment for BPH and concomitant surgical treatment for urethral stricture during the same procedure. BPH surgeries of interest included TURP, HoLEP, laser vaporisation, electrovaporisation, ThuLEP, prostate incision, water ablation, water vapor thermal therapy, prostate stent placement and prostatic urethral lift. Surgical treatment of urethral stricture was defined as internal urethrotomy (OPS code: 5–585), blind dilatation of the urethral stricture (OPS code: 8–139.0), or meatoplasty (OPS code: 5–581). Thus, inclusion required both an N40 and an N35 diagnosis as well as a BPH and a urethral stricture procedure.

The dataset provided information on patient demographics, comorbidities, the year of surgery, and perioperative outcomes. Comorbidities were identified using the ICD-10-GM diagnostic codes. The primary outcome of the present study was to assess the incidence and trends of the management of urethral strictures during BPH surgery. Secondary outcomes included comparisons between patients with urethral stricture versus those with no urethral stricture in terms of perioperative outcomes (urinary retention, incontinence, and reintervention).

### Statistical analysis

Continuous variables were summarized using medians and interquartile ranges (IQR), while categorical variables were presented as absolute numbers and percentages. Multivariable regression analysis was applied to evaluate the perioperative outcomes (urinary retention, incontinence, and reintervention) of patients with versus no urethral stricture. These models were adjusted for age, diabetes, hypertension, and obesity. The results of the multivariable analysis were presented as odds ratios (ORs) with 95% confidence intervals (CIs), and p-values lower than 0.05 were considered statistically significant. All perioperative outcomes were assessed only during hospital stay, and reinterventions were defined as any endourological procedure requiring anesthesia. Data extraction and patient selection were conducted by the Research Data Center using SAS. Subsequent statistical analyses were performed using R scripts developed by our research team (source: Research Data Center, DRG Statistics 2005–2022).

## Results

### Baseline characteristics and trends

Between 2005 and 2022, 1,367,065 patients underwent surgical treatment for BPH in Germany. Among them, 179,272 patients (13%) had a concomitant diagnosis of urethral stricture, while 1,187,793 (87%) had no documented urethral stricture. The median age was the same in both groups at 72 years (IQR: 66–78). Patients with urethral stricture presented lower rates of obesity (4.4% versus 5.3%), hypertension (42% versus 53%), and diabetes (15% versus 18%) compared to those without urethral stricture. The proportion of patients with urethral stricture varied across surgical modalities, with the highest proportion observed in prostate incision (30%) and lower proportions in minimally invasive treatments such as prostatic urethral lift (2%) and water ablation (3%). The baseline characteristics of patients with urethral stricture versus those without urethral stricture during BPH surgery are presented in Table 1.

**Table 1:** Baseline characteristics of patients with urethral stricture versus those without urethral stricture during BPH surgery

Characteristics	No Stricture, n=1,187,793	Stricture**,** n=179,272
Age (years)	72 (66–78)	72 (66–78)
Obesity	62,551 (5.3%)	7813 (4.4%)
Hypertension	634,789 (53%)	75,733 (42%)
Diabetes	218,220 (18%)	26,825 (15%)
*Type of surgery*		
TURP	927,751 (86%)	156,899 (14%)
HoLEP	57,912 (90%)	6413 (10%)
Laser vaporisation	51,915 (89%)	6491 (11%)
Electrovaporisation	22,499 (87%)	3248 (13%)
ThuLEP	13,862 (91%)	1379 (9%)
Prostate incision	5512 (70%)	2361 (30%)
Water ablation	3205 (97%)	94 (3%)
Water vapor thermal therapy	2,59 (90%)	264 (10%)
Prostate stent	1032 (84%)	203 (16%)
Prostatic urethral lift	503 (98%)	8 (2%)
Other	101,143 (98%)	1912 (2%)

Variables are presented as median (interquartile range) or frequencies with proportions

*BPH* benign prostatic hyperplasia, *HoLEP* holmium laser enucleation of the prostate, *ThuLEP* thulium laser enucleation of the prostate, *TURP* transurethral resection of the prostate

Among patients with urethral stricture, internal urethrotomy was the most frequently performed procedure (n = 155,482, 86%), followed by blind dilatation of the urethral stricture (n = 14,162, 9%) and meatoplasty (n = 9,628, 5%). Baseline characteristics and age were largely comparable across these treatment groups. Blind dilatation of the urethral stricture was most commonly performed during prostate stent placement, HoLEP, or ThuLEP, while meatoplasty was most commonly performed during TURP or prostate incision. The baseline characteristics of patients undergoing internal urethrotomy, blind dilatation of the urethral stricture, and meatoplasty during BPH surgery are available in Table 2.

**Table 2:** Baseline characteristics of patients undergoing internal urethrotomy, blind dilatation of the urethral stricture, and meatoplasty during BPH surgery.

Characteristics	Internal urethrotomy, n=155,482	Dilatation of the urethral stricture, n=14,162	Meatoplasty, n=9,628
Age (years)	72 (66–78)	73 (66–79)	71 (65–77)
Obesity	7987 (5.1%)	839 (5.9%)	487 (5.1%)
Hypertension	76,616 (49%)	7817 (55%)	4724 (49%)
Diabetes	26,883 (17%)	2761 (19%)	1791 (19%)
*Type of surgery*			
TURP	137,434 (13%)	11,096 (1.0%)	8369 (0.8%)
HoLEP	4554 (7.1%)	1468 (2.3%)	391 (0.6%)
Laser vaporisation	5592 (9.6%)	488 (0.8%)	411 (0.7%)
Electrovaporisation	2700 (10%)	435 (1.7%)	113 (0.4%)
ThuLEP	1104 (7.2%)	253 (1.7%)	22 (0.1%)
Prostate incision	2210 (28%)	87 (1.1%)	64 (0.8%)
Water ablation	84 (2.5%)	9 (0.3%)	0 (0%)
Water vapor thermal therapy	193 (7.1%)	69 (2.5%)	3 (0.4%)
Prostate stent	159 (13%)	41 (3.3%)	3 (0.2%)
Prostatic urethral lift	5 (1.0%)	3 (0.6%)	0 (0%)
Other	1447 (1.4%)	302 (0.3%)	253 (0.3%)

Variables are presented as median (interquartile range) or frequencies with proportions

*BPH* benign prostatic hyperplasia, *HoLEP* holmium laser enucleation of the prostate, *ThuLEP* thulium laser enucleation of the prostate, *TURP* transurethral resection of the prostate

**Fig. 1:**
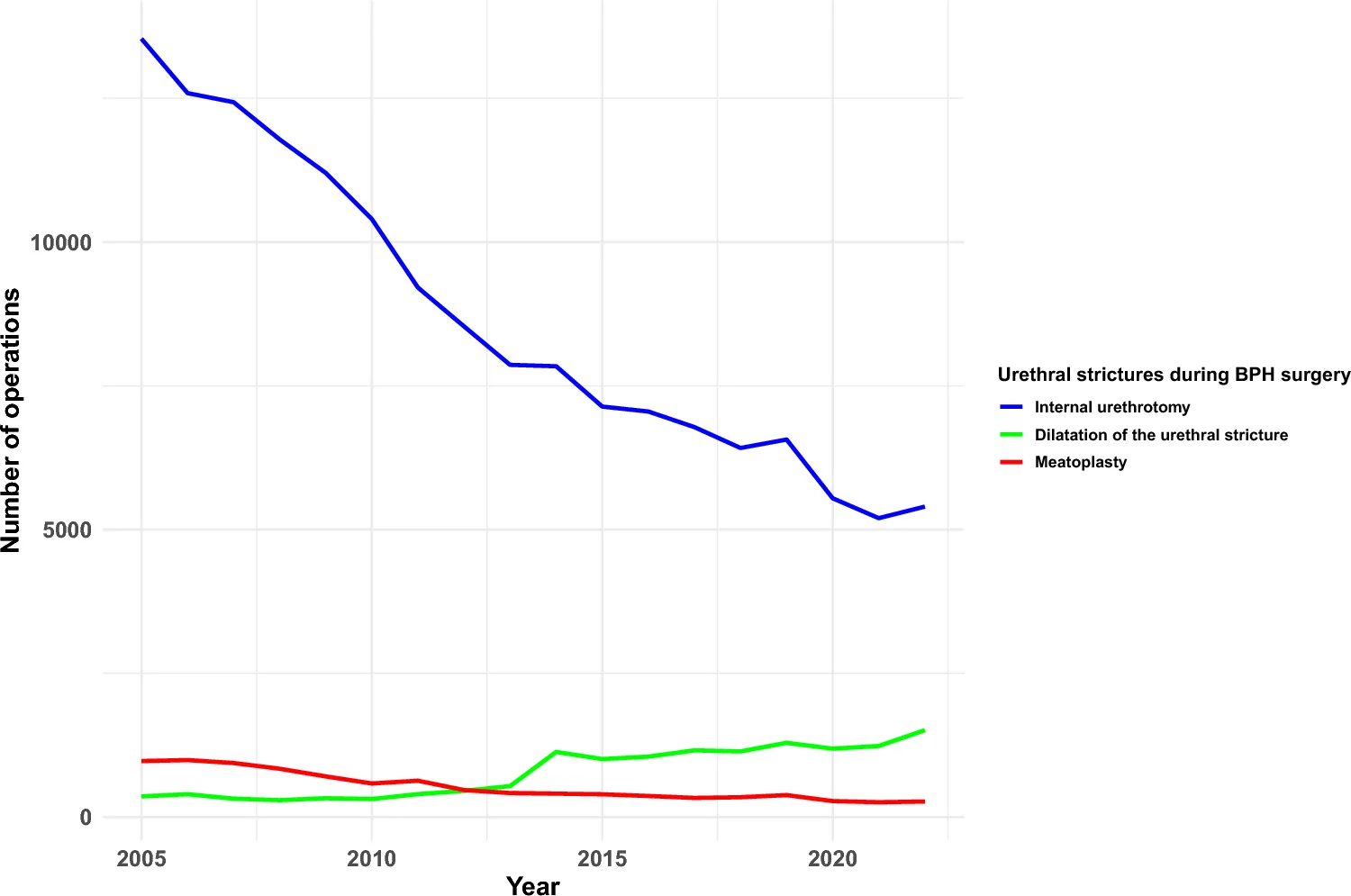
Trends of urethral stricture management techniques during BPH surgery in Germany. BPH: benign prostatic hyperplasia

The annual prevalence of urethral strictures during BPH surgery decreased over the study period, from 14,872 cases in 2005 to 7,184 cases in 2022. Internal urethrotomy remained the predominant treatment modality throughout the study period, although it was performed less frequently over the study period. On the contrary, the use of blind dilatation of the urethral stricture or meatoplasty remained relatively stable within the last few years in Germany. The annual trends of urethral stricture management techniques during BPH surgery are depicted in Fig. 1.

### Perioperative outcomes

Overall, perioperative complication rates were low across the entire cohort. However, patients with a urethral stricture during BPH surgery demonstrated slightly worse outcomes compared to those without stricture in the multivariable analysis. In particular, postoperative urinary incontinence occurred in 6,818 (3.8%) patients with urethral stricture compared to 44,348 (3.7%) in those without stricture (OR: 1.30, 95% CI: 1.26 to 1.33, p < 0.001). Similarly, reoperation rates were higher in patients with urethral stricture, occurring in 1,804 (1%) patients versus 9,372 (0.8%) in those without urethral stricture (OR: 1.52, 95% CI: 1.44 to 1.60, p < 0.001). In contrast, acute urinary retention was less frequent in patients with urethral stricture, occurring in 13,979 (7.8%) patients versus 118,710 (10%) in those without urethral stricture (OR: 0.92, 95% CI: 0.9 to 0.94, p < 0.001).

When stratified by stricture management technique, some variability in perioperative outcomes was observed. Patients undergoing blind dilatation of the urethral stricture exhibited higher rates of urinary retention (1,762 patients; 12%) and incontinence (690 patients; 4.9%) compared to those undergoing internal urethrotomy (14,424 patients; 9.3% and 6,450 patients; 4.1%, respectively) or meatoplasty (913 patients; 9.5% and 403 patients; 4.2%, respectively). Reoperation rates were higher after blind dilatation of the urethral stricture (181 patients; 1.3%) and lower after meatoplasty (57 patients; 0.5%) compared to internal urethrotomy (1,780 patients; 1.1%).

## Discussion

The present nationwide analysis from Germany provides comprehensive insights into the prevalence, management, and perioperative outcomes of concomitant urethral strictures during BPH surgery. Our findings demonstrate that urethral strictures are present in a considerable proportion of patients, affecting approximately 13% of all BPH surgeries. Internal urethrotomy represents the predominant treatment modality, while blind dilatation of the urethral stricture and meatoplasty are less frequently performed. Interestingly, patients with urethral strictures displayed slightly more favorable baseline characteristics compared to those without strictures. Nevertheless, the distribution of strictures varied across surgical techniques, with higher proportions observed in procedures such as prostate incision and lower proportions in minimally invasive treatments. Despite the relatively low overall complication rates, the presence of a urethral stricture during BPH surgery was associated with modestly worse perioperative outcomes. Specifically, patients with urethral strictures experienced slightly higher rates of postoperative incontinence and reintervention, while acute urinary retention was less frequent in this group during hospital stay. Still, it is not clear whether these findings are clinically meaningful, since the absolute differences between the two groups were very small. Notably, the prevalence of urethral strictures during BPH surgery has declined over time, potentially reflecting improvements in diagnostics, surgical techniques, and instrumentation.

A better understanding of urethral strictures during BPH surgery is essential for optimizing preoperative assessment, surgical planning, and patient counseling [17]. Our findings highlight that a considerable number of patients present with concomitant urethral stricture during BPH surgery, necessitating additional diagnostic and therapeutic considerations [18]. Still, drug (paclitaxel)-coated balloon dilatation or urethroplasty is reserved for recurrent or persistent urethral strictures and cannot be performed during BPH surgery [19]. Importantly, urethral strictures appear to have only a modest (probably not clinically relevant) impact on perioperative outcomes, despite their relatively high prevalence during BPH surgery [20]. It seems that the overall complication rates remain low, underscoring the general safety of contemporary surgical management for BPH, even in the presence of concomitant urethral strictures.

Our findings are supported by the results of a study published by our research group on surgical trends of urethral strictures [21]. In that study, internal urethrotomy was identified as the predominant treatment modality, accounting for more than 80% of all procedures, despite a gradual decline in its use over time. Similarly, our analysis confirms the central role of internal urethrotomy as a concomitant treatment modality during BPH surgery, reflecting its technical simplicity and widespread availability. Still, it should be highlighted that this database does not provide details such as stricture location, length, cause, or whether the stricture was primary or recurrent. Moreover, recurrence rates after hospital discharge are also not available. Of note, the perioperative complications of internal urethrotomy, urethral dilatation, and meatoplasty were comparable among patients undergoing correction of the urethral stricture only and those undergoing concomitant correction of the urethral stricture during BPH surgery.

This study represents, to the best of our knowledge, the largest descriptive analysis of the incidence of urethral strictures in patients undergoing BPH surgery. Nevertheless, several important limitations should be acknowledged. Firstly, the retrospective design and the reliance on billing data may have introduced potential inaccuracies due to coding errors or misclassifications. For example, the diagnosis of urethral strictures was based on coding information. Therefore, the true prevalence of urethral strictures in patients undergoing surgery for BPH might be even higher. Additionally, there was a lack of detailed clinical information about prostate volume, length, and location of the urethral stricture, occurrence of multiple urethral strictures, cause of the stricture, operative time, patient laboratory findings (including PSA levels), prior surgeries and interventions (including cystoscopy and urography), as well as detailed patient comorbidity profiles. It should be stated that it was beyond the scope of the present study to compare perioperative outcomes among the different BPH surgical techniques, as this has been the focus of previous studies published by our research group [6, 22, 23]. Of note, the absence of long-term information, functional outcomes (including IPSS, post-void residual, and Qmax), recurrence rates, and patient quality of life further limits the conclusions of this study. Finally, given that the findings of the present study are specific to the German healthcare system, caution is warranted in extrapolating these results to countries with different healthcare systems and surgical practices.

## Conclusions

The present large-scale, real-world analysis demonstrates that urethral strictures are frequently encountered and concomitantly managed during surgery for BPH. Moreover, the overall perioperative complication rates in these cases remain low. Importantly, our findings highlight a declining trend in the prevalence of urethral strictures over time, while internal urethrotomy remains the most commonly applied management strategy. Despite these improvements, urethral stricture continues to represent an important factor that should be taken into account during preoperative evaluation and surgical decision-making. Given the limitations inherent to administrative data, the present results should be considered hypothesis-generating and do not reflect any causal relationship. Therefore, further studies are warranted to better define optimal management strategies and their impact on both perioperative and long-term outcomes.

## Data Availability

No datasets were generated or analysed during the current study.
